# Experience in the Management of Complex Apple Peel Intestinal Atresia in a Limited-Resource Health Center in Mexico

**DOI:** 10.7759/cureus.57446

**Published:** 2024-04-02

**Authors:** José R Rico-Tafoya, Guillermo J Serrano-Meneses, Martín A Serrano-Meneses, Víctor H Portugal-Moreno

**Affiliations:** 1 Neonatal Surgery, Hospital Pediátrico Moctezuma, Mexico City, MEX; 2 Department of Chemical and Biological Sciences, Universidad de las Américas Puebla, Cholula, MEX

**Keywords:** multiple intestinal atresia, tapering enteroplasty, end-to-end anastomoses, apple peel atresia, intestinal atresia

## Abstract

Surgical treatment of complex intestinal atresia is challenging. Moreover, multiple surgical techniques have been described to treat these congenital malformations. As no single/universal technique is useful for every patient, individualized surgical treatment for these complex cases is mandatory. Isolated apple peel atresia (type IIIb), in coexistence with other types of atresia, is a rare event with a poor functional prognosis, which is difficult to treat surgically. Furthermore, the ability to achieve good surgical results becomes more difficult in resource-limited health facilities, such as the Hospital Pediatrico Moctezuma (Mexico City). The objective of this case report of two full-term female newborns with isolated apple peel atresia and an apple peel malformation with distal type IV atresia is to describe the successful surgical technique used in these patients and how to deal with certain postsurgical complications.

## Introduction

Intestinal atresia is a common cause of intestinal obstruction in newborns, with an incidence ranging from 1 in 330 and 1 in 400 live births to 1 in 1,500 and 1 in 3,000 live births [1,2]. Jejunoileal atresia has been classified into four categories, as well as a further subdivision type III (IIIa and IIIb). Among these, IIIb (i.e., apple peel atresia), as well as type IV (i.e., multiple atresias) are uncommon types and are reported to have the highest morbidity and mortality rates [1].

Apple peel atresia consists of a proximal dilated bowel and a distal segment spiraling around its blood supply, both disconnected from each other [2]. Only a few cases of apple peel atresia coexisting with multiple distal atresia have been reported in Latin America. Furthermore, the surgical complexity and the resources needed (i.e., neonatal intensive care unit, total parenteral nutrition, appropriate suture materials, linear staplers, neonatal ventilators, and pediatric anesthesiologists) for successful management further complicate the prognosis of these newborns in developing countries.

The objective of this case report of two full-term female newborns with isolated apple peel atresia and an apple peel malformation with distal type IV atresia is to describe the successful surgical technique used in these patients and how to deal with certain postsurgical complications.

## Case presentation

Case 1

**Figure 1:**
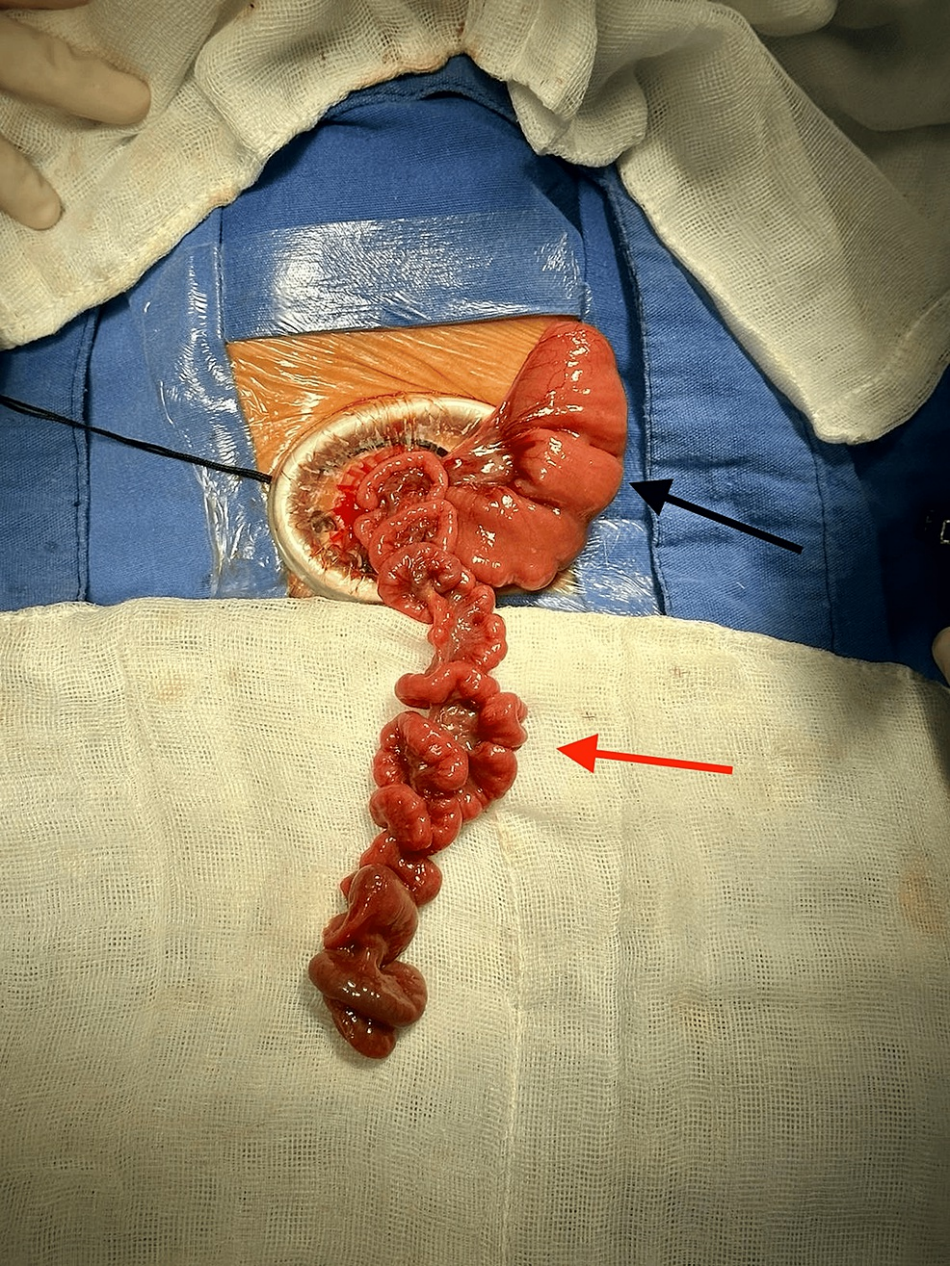
Proximal dilated bowel near the ligament of Treitz (black arrow). Apple peel atresia (red arrow).

A three-day-old female newborn was delivered by cesarean section with a gestational age of 40 weeks and a birth weight of 2,650 g. She was admitted to the newborn intensive care unit with the diagnosis of intestinal obstruction. A periumbilical incision was performed using an Alexis (Applied Medical Resources Corporation, Rancho Santa Margarita, CA) wound protector/retractor. An apple peel intestinal atresia (IIIb) was observed (Figure 1). The dilated bowel was 20 cm long from the Treitz ligament to the atretic site, while the length of the distal segment was 40 cm long. The size discrepancy between proximal and distal bowel was 8:1.

**Figure 2:**
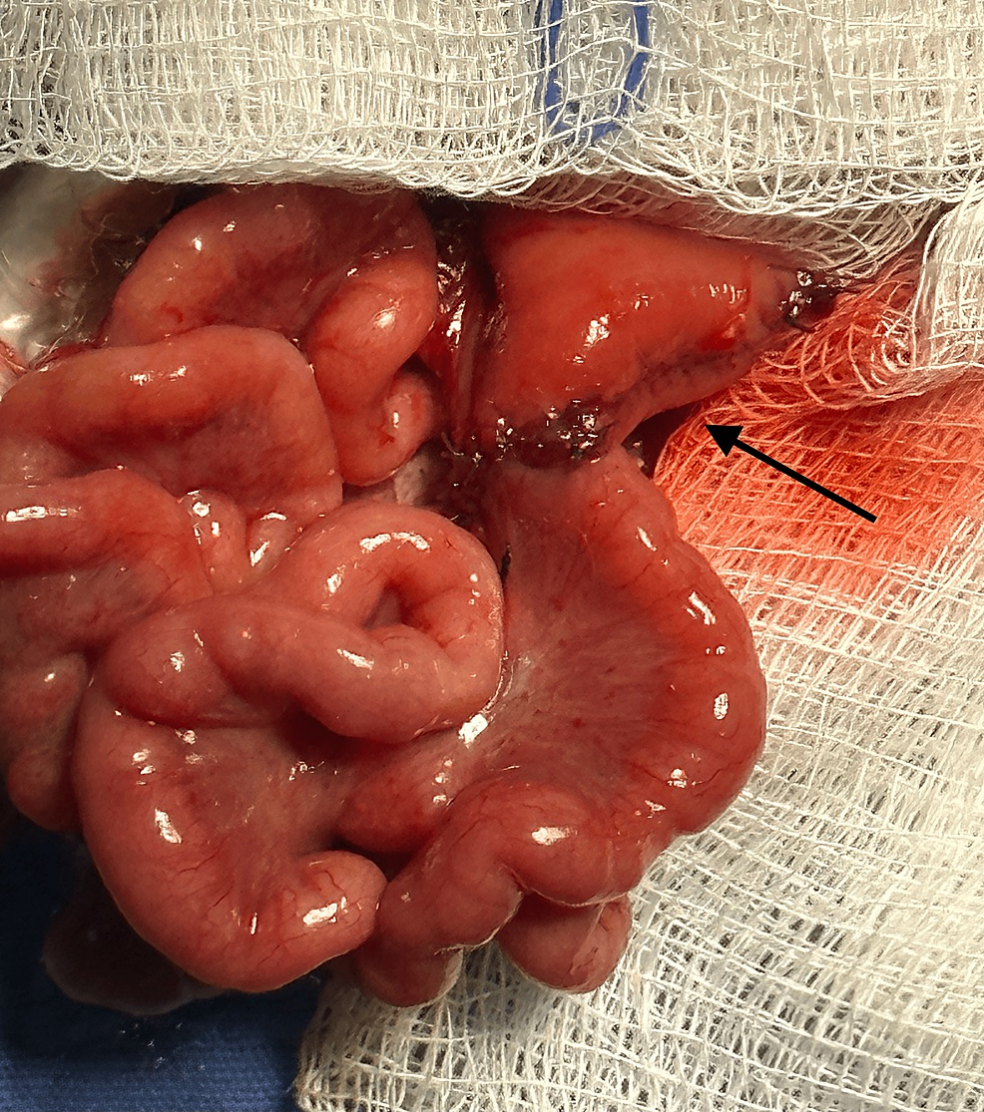
First attempt of a tapering enteroplasty with a short length, which evolved into a blind loop syndrome (arrow).

**Figure 3:**
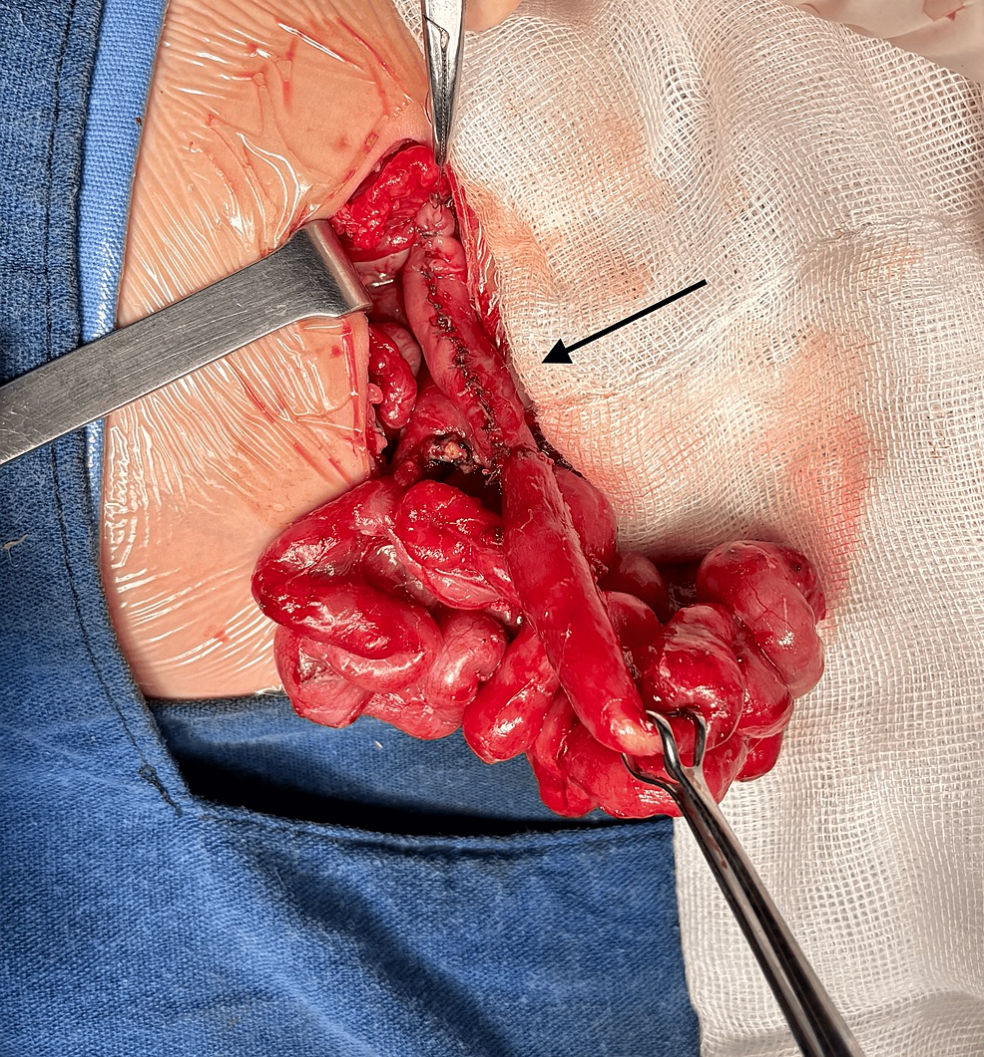
Second and successful attempt of tapering enteroplasty achieved through a longest proximal segment reduction. Note there is no size discrepancy between the proximal and distal bowel (arrow).

We performed a tapering enteroplasty of the proximal dilated bowel with a 5-0 polyglactin 910, 17 mm taper point needle, with a simple interrupted suture (single layer); a 2:1 discrepancy between the proximal and distal bowel and a short length tapering was observed (Figure 2). After a single-layer end-to-end anastomosis, the stability of this procedure was verified with hermeticity/permeability tests. Postoperatively, it was not until day seven that we identified adequate distal passage of intestinal gas on abdominal X-ray films and decided to initiate enteral feeding, which was not successful. A prokinetic was added to the management of the newborn. On day 16, we attempted enteral feeding again by continuous infusion of milk formula, but we were unsuccessful. An abdominal X-ray showed dilation in the proximal bowel. Thus, on day 20, an open laparotomy was performed due to suspicion of dysfunctional anastomosis. An inadequate passage of intestinal content from the proximal to the distal bowel was evidenced. A new longer tapering enteroplasty (7 cm) of the proximal bowel and an end-to-end re-anastomosis was accomplished (Figure 3). On day eight, after the second surgical procedure, a new feeding attempt was successful through continuous infusion of milk formula, and on day 14, complete oral feeding was achieved. The patient was successfully discharged from the hospital on day 41.

Case 2

**Figure 4:**
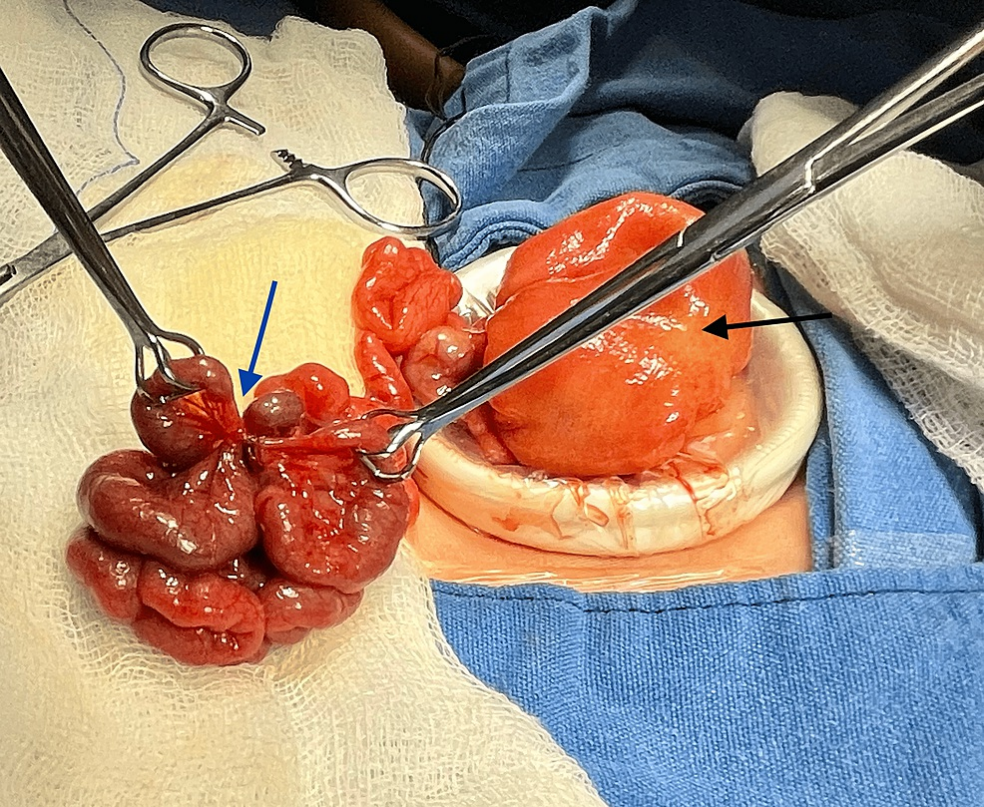
Proximal dilated bowel (black arrow). Apple peel atresia. Type IV atresia distal (two atresias in V-shaped mesentery) (blue arrow).

A one-day-old female newborn was delivered by cesarean section with a gestational age of 38 weeks and a birth weight of 2,510 g. She was admitted to the newborn intensive care unit with the diagnosis of intestinal obstruction. A periumbilical incision was performed using an Alexis (Applied Medical Resources Corporation, Rancho Santa Margarita, CA) wound protector/retractor. An apple peel intestinal atresia (IIIb) was observed (Figure 4). The proximal jejunum was 15 cm long, from the ligament of Treitz to the atretic site with a size discrepancy of 10:1; the length of the distal bowel (20 cm) was compromised by a coexisting type IV atresia (two atresias with a V-shaped mesenteric gap).

**Figure 5:**
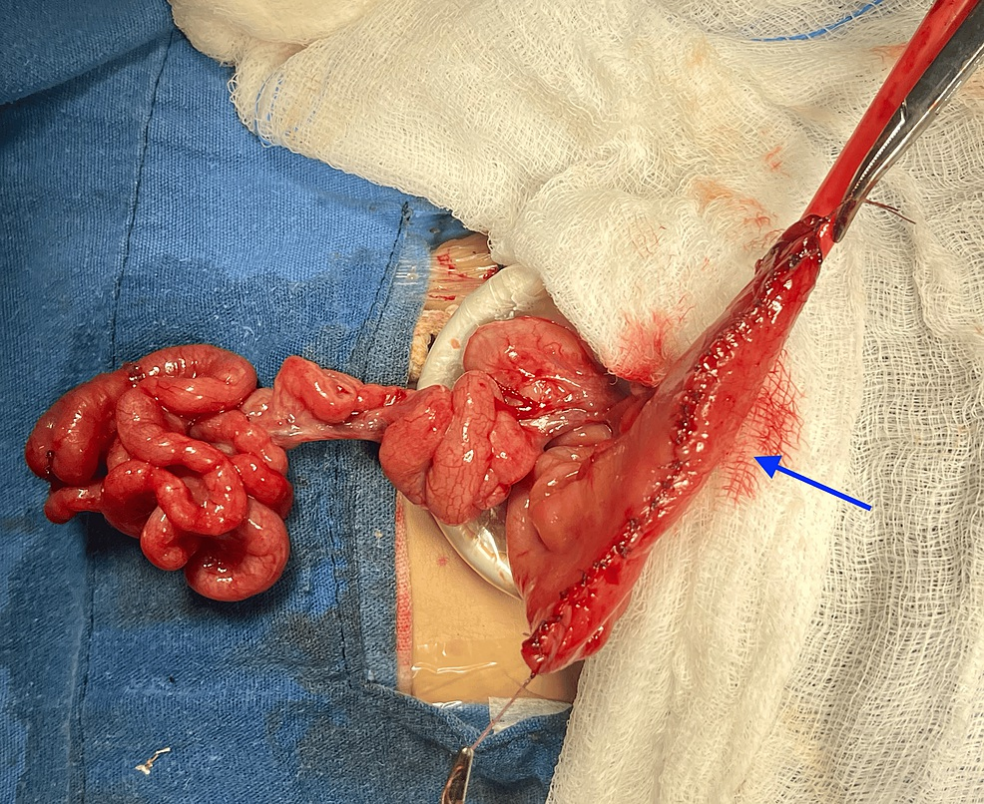
Tapering enteroplasty with sufficient length and caliber to perform a 1:1 single-layer end-to-end anastomosis (arrow).

**Figure 6:**
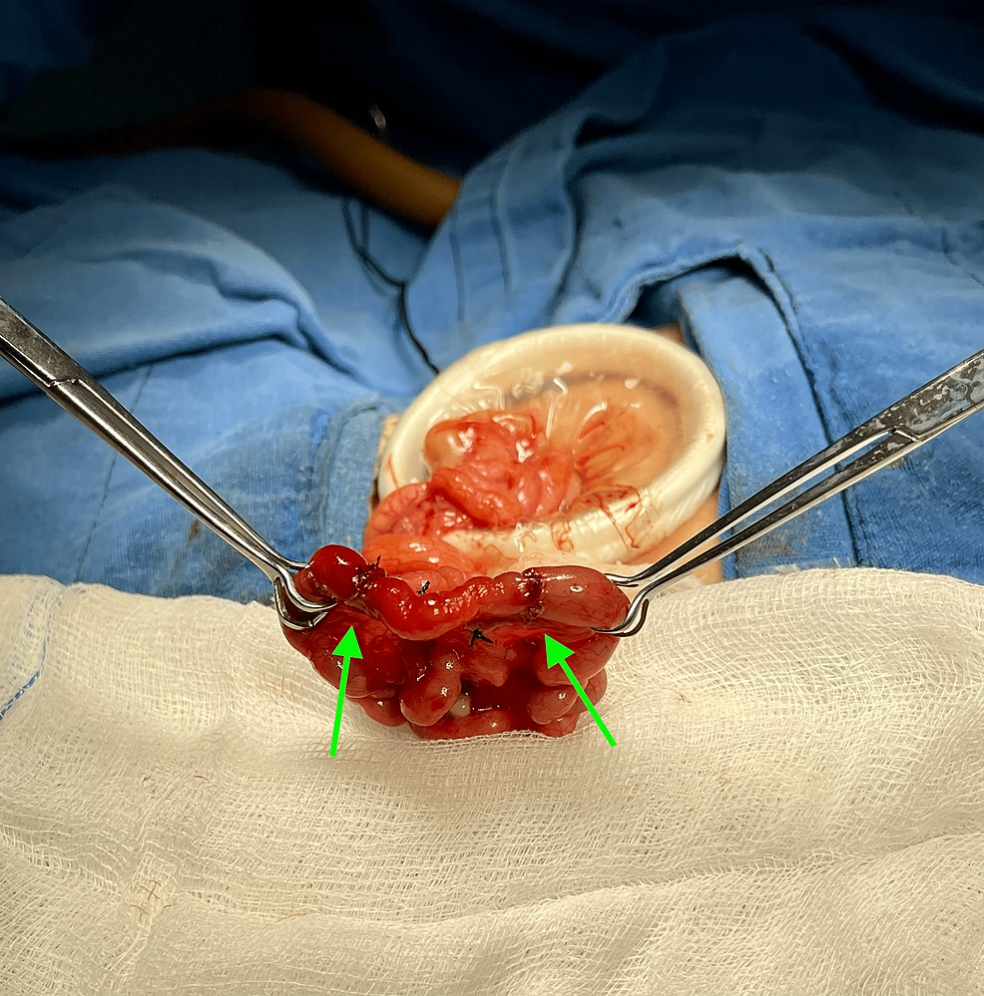
Two single-layer end-to-end anastomoses made to preserve intestinal length (arrows).

A tapering enteroplasty of the proximal dilated bowel with a 5-0 polyglactin 910, 17 mm taper point needle with a single-layer simple interrupted suture was performed (Figure 5). After this procedure, three single-layer, end-to-end anastomoses were achieved; two of them were distal to the dilated bowel to preserve intestinal length (Figure 6). The postoperative period was uneventful, and total enteral feeding was achieved on day 24 after the operation. She was discharged from the hospital on day 25.

## Discussion

The mortality rate of complex intestinal atresias has been historically high. However, in the last few years, the survival rate has increased significantly in developed countries, and the mortality rate associated with intestinal atresia has been reduced from 30-50% to 10% [3,4]. To our knowledge, there are no published survival analyses of these children in Mexico or Latin America; however, the Global PaedSurg Research Collaboration reported an all-cause, in-hospital mortality rate of intestinal atresia of 60% to 21.4% for low and middle-income countries, respectively (Mexico included) and 3.3% for high-income countries [5]. Apple peel atresia and multiple complex atresias still represent a heavy burden of morbidity and mortality in Mexico, mainly because of their complex nature but also because of the limited resources at most health facilities throughout the country.

In both cases presented here, the complexity in the surgical management was the proximity of the atresia to the ligament of Treitz, the disparity of the proximal and distal ends, foreshortened bowel, and a large mesenteric gap. Therefore, a crucial step is to find the means to adjust the diameter to perform a primary reconstruction. At our local institution, we do not have access to linear staplers, which have been reported to significantly shorten the operative time, improve the rapid onset of peristalsis, make early feeds possible, and reduce the time of hospital stay [6,7]; thus, the solution we found was to perform a hand-sewn reduction tapering of the proximal segment. This procedure has several advantages: it reduces the diameter of the proximal dilated bowel to adapt to the small and narrow distal intestinal segment, it accelerates fluids through the anastomosis, reduces stasis and, therefore, the risk of bacterial translocation, and significantly reduces septicemia [8]. Through this process, as we learned in the first presented case, a long reduction tapering of the proximal segment (at least 5 cm) is needed to reduce the risk of blind loop syndrome and prevent the need for subsequent surgical interventions. Moreover, the starting of the enteral feeding was difficult, but supplying the milk formula in a continuous infusion, combined with the use of prokinetics, was a successful strategy to achieve the goal of complete oral feeding. We delayed the second surgical procedure as proximal dilated bowel walls do not adequately coapt, and peristalsis is incapable of producing an adequate upstream pressure gradient [9]. We wrongly assumed that this effect could be reduced by a physiological adaptation leading to a recovery of motility of the proximal segment over time.

When treating these patients, there is always the need to preserve the length of the intestine at all costs, as most of these children have less than half of the normal length of the small bowel and have a physiologically short bowel [9]. For this reason, in the second case, we performed three primary end-to-end anastomosis and a proximal reduction tapering long enough to avoid a blind bowel syndrome. This allowed us to begin enteral feeding earlier than in the first patient and always through oral feeding. In this case, the progression of the total oral feeding volume was not achieved until day 24, mainly due to the fear of a clinical evolution similar to that of the first case; however, the experience learned in this case will help us initiate oral intake earlier and a more quick establishment of full oral feeding in future patients.

A significant factor contributing to the prognosis of patients with complex intestinal atresias are the following components of neonatal surgical care that are considered essential in high-income settings (not all of them accomplished in our hospital): birth at a pediatric surgery center, effective resuscitation, timely ambulance transfer born in or referred to district hospitals, use of a surgical safety checklist, and basic neonatal intensive care unit resources such as ventilation, central intravenous access, and parenteral nutrition [5]. In some countries, the lack of appropriate resources forces surgeons to abandon any attempt at surgical correction [10] even when surgery remains the mainstay of therapy for these children. It has been acknowledged that patients in low and middle-income countries traveled further from home to the study hospital; had higher proportions of patients presenting with hypothermia, sepsis, and hypovolemia; and had a higher proportion of patients not receiving surgical intervention [5]. Moreover, patients with apple peel atresia who survive the initial operative and postoperative period are likely to experience normal bowel function with adequate growth and development [11].

The main limitation of this study is that we are only reporting the experience of two isolated cases, therefore, a long-term prospective study including more patients treated with this technique is required to objectively evaluate the advantages and disadvantages of this alternative surgical technique for limited-resource health facilities.

## Conclusions

We highlight the importance of publishing our experience when treating these complex cases despite the lack of resources and facilities compared to those in developed countries. We suggest that the techniques presented here can be extrapolated to other institutions like ours, both locally and abroad. A long-term prospective study using this technique is required to objectively evaluate its advantages and disadvantages.
